# A Crispy Diet: Grazers of *Achromatium oxaliferum* in Lake Stechlin Sediments

**DOI:** 10.1007/s00248-018-1158-4

**Published:** 2018-02-28

**Authors:** Sina Schorn, Heribert Cypionka

**Affiliations:** 1 Institute for Chemistry and Biology of the Marine Environment (ICBM), Carl-von-Ossietzky University of Oldenburg, Carl-von-Ossietzky-Str. 9-11, 26129 Oldenburg, Germany; 2 Present Address: Max Planck Institute for Marine Microbiology, Bremen, Germany

**Keywords:** Large sulfur bacteria, Plathelminthes, Ciliates, Amoebae, Oligochetes, Aquatic fungi

## Abstract

*Achromatium* is the largest freshwater bacterium known to date and easily recognised by conspicuous calcite bodies filling the cell volume. Members of this genus are highly abundant in diverse aquatic sediments and may account for up to 90% of the bacterial biovolume in the oxic-anoxic interfaces. The high abundance implies that *Achromatium* is either rapidly growing or hardly prone to predation. As *Achromatium* is still uncultivated and does not appear to grow fast, one could assume that the cells might escape predation by their unusual shape and composition. However, we observed various members of the meiofauna grazing or parasitizing on *Achromatium.* By microphotography, we documented amoebae, ciliates, oligochetes and plathelminthes having *Achromatium* cells ingested. Some *Achromatium* cells harboured structures resembling sporangia of parasitic fungi (chytrids) that could be stained with the chitin-specific dye Calcofluor White*.* Many Achromatia carried prokaryotic epibionts in the slime layer surrounding the cells. Their regular distribution over the cell might indicate that they are commensalistic rather than harming their hosts. In conclusion, we report on various interactions of *Achromatium* with the sediment community and show that although *Achromatium* cells are a crispy diet, full of calcite bodies, predators do not spare them.

## Introduction

*Achromatium* is the genus with the largest freshwater bacteria known to date. Single cells with a length of up to 125 μm [1] are visible even with the naked eye. The volume of an *Achromatium* cell exceeds that of a “normal” bacterium by a factor of 10^4^–10^5^ [2]. Like other large sulfur-oxidising bacteria, such as *Beggiatoa* and *Thiomargarita* [3, 4], *Achromatium* cells contain small sulfur globules. In a recent study, we have shown that single *Achromatium* cells harbour multiple DNA spots showing a community-like genome diversity [5]. Phenotypically most conspicuous and unique to *Achromatium* are numerous intracellular calcite bodies (CaCO_3_), which fill major parts of the cell volume [6]. The biological role of these calcite bodies is under debate [7, 8].

*Achromatium* can be found within the oxic-anoxic transition zone in freshwater [2, 6, 9–12], brackish [13], and marine [14] sediments and may reach cell counts of 10^3^–10^5^ cells per cubic centimetre accounting for 90% of the bacterial biovolume in these layers [6, 14, 15]. The high abundance of *Achromatium* implies that the cells are either rapidly growing or not prone to predation. As *Achromatium* is uncultivated exact growth rates are unknown. Mortality factors, such as predation and parasitism, which might reduce natural population sizes of *Achromatium,* have neither been reported. However, for grazers *Achromatium* cells might be unattractive as food source due to the massive amounts of calcite. Thus, we assumed that the cells might escape predation by their unusual size and composition.

During a series of physiological experiments *Achromatium* cells were collected from sediment storages in glass jars and microscopically examined. In doing so, we repeatedly detected grazers that contained ingested *Achromatium* cells and took microphotographs of them. We present here a qualitative description that sheds new light on the ecological relationships of *Achromatium* with the sediment community.

**Fig. 1:**
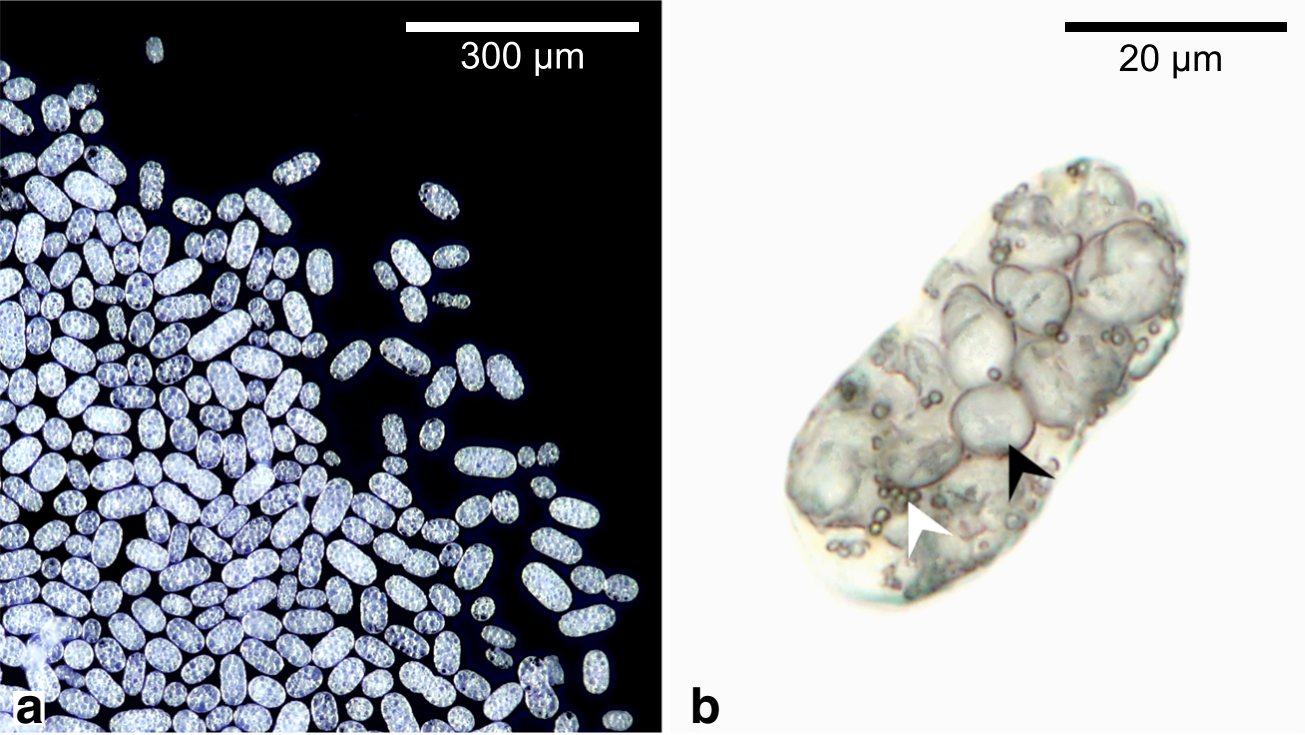
Bacteria of the genus *Achromatium*. **a** Incident light micrograph of manually purified *Achromatium* cells. Light reflections by intracellular inclusions let the cells appear white in front of a black background. **b** Transmitted light image of a single *Achromatium* cell showing large intracellular calcite bodies (black arrow) and small sulfur globules (white arrow)

For our study, sediment samples were taken from Lake Stechlin, an oligotrophic freshwater lake near Neuglobsow, Brandenburg, Germany (53° 9′ 5.59″ N; 13° 1′ 34.22″ E). The sediment samples were either immediately analysed or stored in glass jars at 15 °C with a diurnal 12 h/12 h light/dark cycle. Under these conditions, *Achromatium* cells stayed active over several months. Sediment material was collected from the upper layers (< 1 cm) of the glass jars and studied under an inverted microscope (Zeiss Diavert). *Achromatium* cells appear white in front of a black background (Fig. 1a) due to light reflection by the calcite bodies and sulfur globules (Fig. 1b), which allows to detect them in bulk sediment and even inside of grazers.

To estimate the natural abundance of the grazers in the sediment of Lake Stechlin, we took cores of fresh sediment. The cores were subsequently divided into oxic surface layer (0–3 mm), oxic-anoxic transition zone (6–10 mm) and anoxic layer (10–15 mm). The sediment samples were filtered through an 80-μm mesh to wash out small organisms (e.g. ciliates), the unsieved sediment was analysed for the presence of larger grazers.

Epifluorescence microscopy (Olympus BX51) and Sybr Green I were used to visualize mucous-associated prokaryotes. To study fungal infections, *Achromatium* cells were stained with Calcofluor White, a fluorescent dye used to stain fungal chitin. Pictures were taken with a Canon EOS 600D camera from live samples and further processed with PICOLAY [16].

## Grazers Ingesting *Achromatium* Cells

**Fig. 2:**
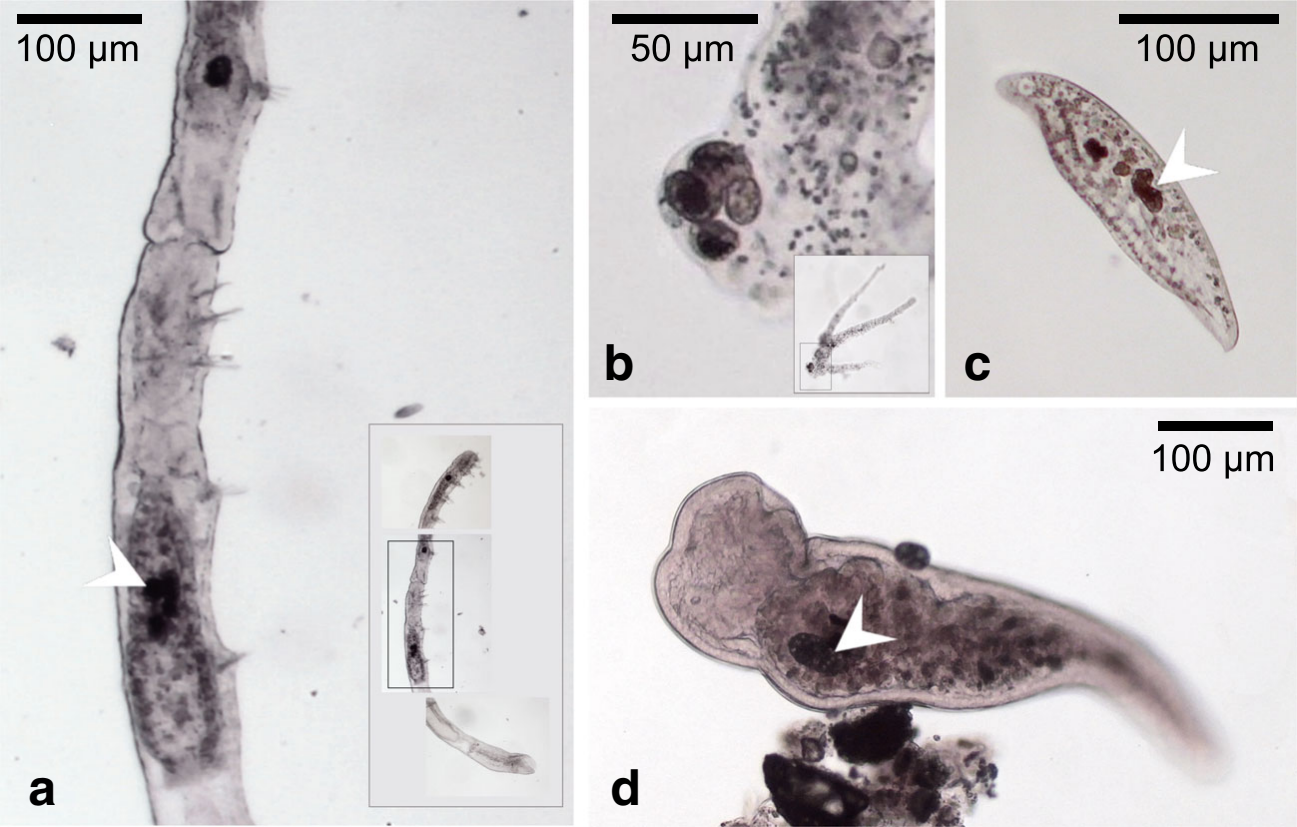
Grazers of *Achromatium*. Ingested *Achromatium* cells (marked with white arrows) were recognised in the transparent body structures of various grazers. **a** Oligochete (resembling *Chaetogaster diastrophus*). **b** Amoeba (resembling *Chaos diffluens*). **c** Ciliate (resembling *Amphileptus*). **d** Plathelminth (resembling *Stenostomum leucops*)

In fresh sediment samples as well as in those stored in glass jars for several months, we observed amoebae, ciliates, oligochetes and plathelminthes having *Achromatium* cells in food vacuoles or intestinal compartments of their transparent body structures. The grazers resembled common inhabitants of freshwater sediments. Most often, we recognised ciliates (resembling *Amphileptus*) with ingested *Achromatium* cells. These ciliates apparently selected the smallest *Achromatium* cells in the population with a length below 20 μm (Fig. 2c). Occasionally, we observed amoebae (resembling *Chaos diffluens*) with ingested *Achromatium* cells in their food vacuoles (Fig. 2b). Furthermore, oligochetes (resembling *Chaetogaster diastrophus,* Fig. 2a) and plathelminthes (resembling *Stenostomum leucops*, Fig. 2d) were observed with several ingested *Achromatium* cells in their digestive tracts. Besides these grazers, larvae of copepods and crustaceae, as well as small snails were present at the sediment surface. Although rather abundant, these organisms were not observed to carry ingested *Achromatium* cells. In fresh sediment we observed grazers in most of the analysed samples, however at fluctuating abundancies, not allowing for proper statistics.

## Fungal Infection

**Fig. 3:**
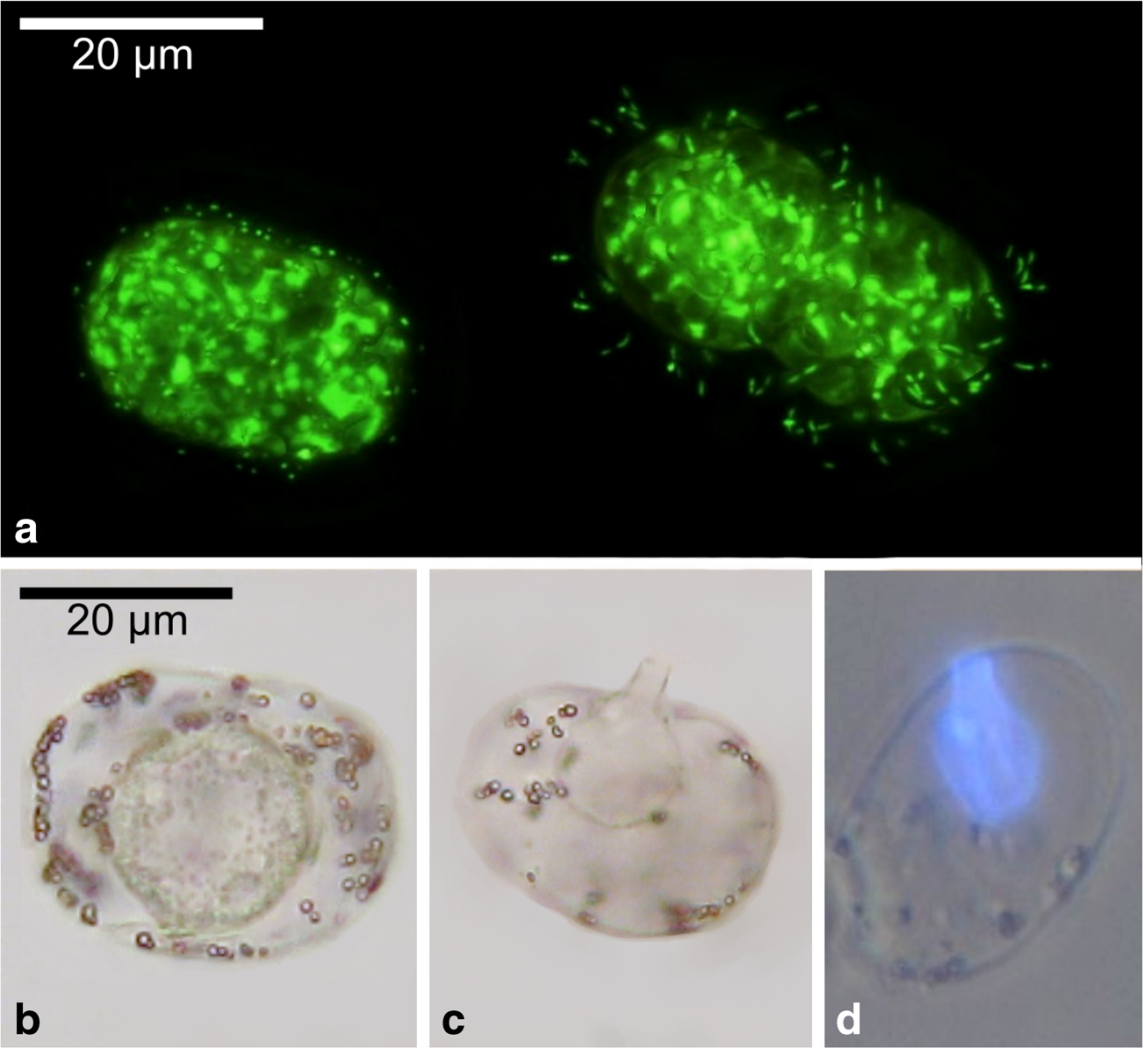
Prokaryotic epibionts and parasitic fungi. **a** Mucus-associated coccoid (left) or rod-shaped (right) bacteria around *Achromatium* cells visualised with Sybr Green I. Focus-stacked image processed with PICOLAY; Original image stack courtesy of H-P. Grossart. **b** Bulb-shaped internal structures resembling zoosporangia of parasitic fungi. **c** Sporangium with tube-shaped funnel. **d** Calcofluor White staining of intracellular chitin-containing structures

Occasionally, we observed *Achromatium* cells having intracellular bulb-shaped structures (Fig. 3b) and tube-shaped funnels (Fig. 3c). Morphologically, these structures resembled zoosporangia of *Chytridiomycota*, a group of aquatic fungi known to parasitize cyanobacteria [17]. Calcofluor White staining confirmed these structures being composed of chitin (Fig. 3). The infected *Achromatium* cells did not contain calcite bodies. The absence of these alone is not an indicator of physiological damage, as we often observed calcite-free mobile cells in the sediment. The infected cells described here, however, were immobile.

## Prokaryotic Epibionts

Many *Achromatium* cells were covered by slime, harbouring prokaryotic epibionts [6, 14], as shown by epifluorescence microscopy with Sybr Green I. Sometimes, a uniform morphotype, either rod-shaped or coccoid cells (Fig. 3a) was dominating on single *Achromatium* cells. The associated bacteria were evenly distributed within the slime matrix indicating a beneficial or commensalistic relation. The formation of micro-colonies, as indication for rapid proliferation during substrate degradation was not observed.

## Discussion

Our observations show that *Achromatium* is exposed to selective pressure through predation and parasitism. Living at the shallow sediment-water interface, or slightly below, *Achromatium* is easily accessible for grazers that are adapted to the interstitial habitat. Although the cells are unusually large and full of calcite bodies, there are a variety of organisms grazing on them. Typically, food vacuoles have a low pH. The calcite present in ingested *Achromatium* cells might buffer this, and digestion will require additional acidification [10]. Thus, acidic digestion compartments can help to overcome the challenges when feeding on such “crispy” bacteria. Whereas calcite has no nutritional value, other cellular components of *Achromatium* such as the surrounding mucous matrix and the epibionts might represent an attractive food source for grazers. Although our samples, derived from fresh sediment as well as from stored sediment of varying ages, were not suited for a quantitative analysis, we assume that grazers from several groups control the population density of *Achromatium* in its natural habitat. The observed grazers resembled common organisms of freshwater environments [18, 19]. As these grazers do not seem to be specialised to feed on *Achromatium* a similar grazing pressure might affect also brackish and marine populations. Fungal infection might be an additional mortality factor for natural populations. It was previously shown that cyanobacteria are infected by parasitizing chytrids [17]. Given their widespread abundance in freshwater and marine environments it is likely that also *Achromatium* is a host for them.

Therefore, our initial hypothesis that *Achromatium* is not prone to predation has to be rejected. Instead, they are exposed to considerable pressure from parasites and grazers and have to compensate this with a corresponding growth rate.
